# Delegation of GP-home visits to qualified practice assistants: assessment of economic effects in an ambulatory healthcare centre

**DOI:** 10.1186/1472-6963-10-155

**Published:** 2010-06-08

**Authors:** Neeltje van den Berg, Claudia Meinke, Melanie Matzke, Romy Heymann, Steffen Fleßa, Wolfgang Hoffmann

**Affiliations:** 1 Institute for Community Medicine, University of Greifswald, Ellernholzstraße 1-2, 17487, Greifswald, Germany; 2 Faculty of Law and Economics, University of Greifswald, Friedrich-Loeffler-Straße 70, 17489 Greifswald, Germany

## Abstract

**Background:**

Against the background of a decreasing number of general practitioners (GPs) in rural regions in Germany, the AGnES-concept (AGnES = GP-supporting, community-based, e-health-assisted, systemic intervention) supports the delegation of regular GP-home visits to qualified practice assistants. The concept was implemented and evaluated in different model projects in Germany.

To explore the economic effects of this concept, the development of the number of home visits in an ambulatory healthcare centre was analysed and compared with the number of home visits in the surrounding county.

**Methods:**

Information about GP-home visits was derived from reimbursement data of the ambulatory healthcare centre and a statutory health insurance. Information about home visits conducted by AGnES-practice assistants was collected from the project documentation over a time period of 12 consecutive quarter years, four quarter years before the beginning of the project and 8 quarter years while the project was implemented, considering background temporal trends on the population level in the study region.

**Results:**

Within the ambulatory healthcare centre, the home visits by the GPs significantly decreased, especially the number of medically urgent home visits. However, the overall rate of home visits (conducted by the GPs and the AGnES-practice assistants together) did not change significantly after implementation of the AGnES-concept. In the surrounding county, the home visit rates of the GPs were continuous; the temporal patterns were approximately equal for both usual and urgent home visits.

**Conclusion:**

The results of the analyses show that the support by AGnES-practice assistants led to a decrease of GP-home visits rather than an induction of additional home visits by the AGnES-practice assistants. The most extended effect is related to the medically urgent home visits rather than to the usual home visits.

## Background

Due to changes in the demographical structure of the population, especially in rural areas in Germany, the number of patients will increase, specifically for age-associated chronic diseases and multi-morbidity [1,2]. The age-related increase of morbidity and decrease of mobility will probably lead to an increasing need for GP-home visits.

The development of the age structure of the general practitioners (GPs) corresponds to the total population of Germany. More than 20% of the GPs in Germany are 60 years or older [3]. Especially in rural regions in eastern Germany, the succession of retired GPs is difficult. Local gaps in primary healthcare are already existent and can be expected to increase over the next few years.

In Germany, GPs carry on the most important part of primary care. Other medical professions, for example nurses, have no significant role in the structural organization of primary health care. However, the GP has the opportunity to delegate activities to qualified practice employees (nurses or physician assistants). Traditionally, delegation was restricted to a limited array of specific tasks. Liability issues and insufficient reimbursement for home visits by GP-practice employees have further limited delegation as an option in primary health care.

In the context of these challenges, the AGnES-concept (AGnES = GP-supporting, community-based, e-health-assisted, systemic intervention) was developed. The AGnES-concept allows the GPs an extended delegation of medical activities, especially in the context of home visits, to qualified GP-practice employees (AGnES-practice assistants). The concept was developed specifically for sparsely populated regions with an imminent or already existing undersupply with GPs. Its main goal is to enable the remaining GPs to assure medical care for more patients and in a larger area to compensate for practices which are discontinued after the GPs' retirement [4,5].

The AGnES-concept of delegation of GP-home visits and the associated model projects triggered a broad discussion among professional organisations of physicians and nurses, statutory health insurances, and politicians about chances and possibilities, legal limitations, and acceptance of this concept by patients and physicians.

In many other countries, models to integrate other medical professions into primary care are more common. Extended options for delegation up to the substitution of core GP-activities to qualified nurses are implemented and evaluated in various countries with mainly favourable results [6-10]. Other concepts include structured home visiting programmes for elderly people [11,12]. In some of these programmes home visits include special modules like falls prevention and case management.

Seven AGnES-model projects in four federal states of Germany were carried out between the years 2005 and 2008. In total, 11 228 home visits were conducted involving 1 430 mostly multimorbid patients with an average age of 78.6 years. About 300 different delegated activities were documented, varying from the measurement of diagnostic parameters (e.g. blood pressure, blood glucose, peakflow) to advising on a variety of health-related issues, and medical tasks like taking of blood samples, injections and wound care [4,5].

The acceptance of the concept among patients was high: 94% of the patients reported that AGnES-practice assistants can conduct home visits and address special topics (for example falls prevention, geriatric assessment, and telemedicine [13,14]) and the GP conducts only home visits in cases of medical urgency [15].

The results of a standardized paper-and-pencil interview of the participating GPs showed that 90% of the GPs found that the AGnES-concept reduced their workload. All participating GPs found that the quality of care within the AGnES-concept was comparable to usual care for the majority of the participating patients [5].

### Research questions

This paper focuses on the effect of delegating GP-home visits to qualified practice assistants on the number of home visits. The central question is: did the implementation of the AGnES-concept actually reduce the workload of the GPs? This question is addressed by three research questions:

- Did the number of GP-home home visits decrease during the project?

- Were there different effects considering usual and medically urgent GP-home visits separately?

- Did the total number of home visits (sum of GP-home visits and delegated home visits) increase during the project time period?

Setting for the analysis was an ambulatory healthcare centre in the county Oberspreewald-Lausitz in the Federal State of Brandenburg (north-eastern Germany) which participated in the AGnES-Brandenburg project from July 2006 until December 2008. Here, three AGnES-practice assistants were working full-time for a total of six GPs.

The county Oberspreewald-Lausitz is a rural county with some small cities and villages. 72 GPs have their practices in this county. The ambulatory healthcare centre is located in one of these small cities (Lübbenau). The six GPs in the ambulatory healthcare centre are, together with four more GPs in practices in the city of Lübbenau, responsible for primary care in Lübbenau and its environs. The three practice assistants were nurses with a large professional experience.

This setting allowed a comprehensive analysis of the effect of the option to delegate home visits. In an ambulatory healthcare centre with six GPs, it is likely that they will represent each other. This can be important to correct eventual seasonal effects in the home visit rates due to longer periods of absence of particular GPs.

To obtain a more precise picture of the effects of the intervention, both usual and medically urgent GP-home visits were analysed.

## Methods

The analyses of the development of the number of home visits were conducted on the basis of primary data from the AGnES-project and secondary data from the reimbursement data of the ambulatory healthcare centre. All calculations were based on consecutive three-months-periods (quarter years), corresponding to the reimbursement-calculations for the Association of Statutory Health Insurance Physicians. The analyses were conducted for a time period of 12 consecutive quarter years starting with the third quarter year of 2005 and ending in the second quarter year of 2008. The first four quarter years covered the time before the beginning of the project.

Three different data sets were used:

1. Reimbursement data of all patients of the ambulatory healthcare centre, insured with any of the German statutory health insurances. More than 95% of the patients of the ambulatory healthcare centre are statutory insured.

In the data set for the first analysis, the GP-home visits were categorized by medical urgency:

- usual (not urgent) home visits in a private household;

- usual home visits in a private household immediately after another home visit (urgent or usual) in the same household, e.g. to treat another family member or home visits in retirement or nursing homes;

- urgent home visits outside practice consultation-hours including bank holidays;

- urgent home visits within practice consultation-hours or at night.

These classes are the standard categories, used for reimbursement by the statutory health insurances. We combined these categories into two main classes: usual and urgent home visits.

2. Data of the statutory health insurance AOK (Allgemeine Ortskrankenkasse, engl.: General Regional Health Insurance) Brandenburg.

For the second analysis, the subgroup of patients, insured by the large statutory health insurance AOK Brandenburg was used. Here, also comparative data from the county (Landkreis) in which the health care centre is located (county Oberspreewald-Lausitz) was available. For the county, only aggregated data for the total group of patients were available.

The home visits data were categorized according to the same categories as the dataset under point 1.

The data of the county Oberspreewald-Lausitz was used to compare the development of the number of home visits of the ambulatory healthcare centre with a comparable region without an intervention to exclude possible systemic changes during the project.

3. The number of home visits conducted by AGnES-practice assistants was derived from the project documentation [5].

For all data sets, the absolute number of home visits was standardized to the number of home visits per 1000 patients.

Differences in the number of home visits between 4 consecutive quarter years before the start of the project and 8 consecutive quarter years during the project were statistically analysed using the nonparametric Wilcoxon rank-sum test for both the patients of the ambulatory healthcare centre and of the whole county for usual and medically urgent GP-home visits, AGnES-home visits and the total number of home visits.

## Results

The main characteristics of the project AGnES-Brandenburg are shown in Table 1.

**Table 1:** Characteristics of the AGnES model project in the Federal State of Brandenburg

**Number of participating GPs (n)**	6
**Number of AGnES-practice assistants (n full time equaivalents)**	3
**Number of patients (n)**	379
**Women (n)**	244
**Men (n)**	135
**Age, mean (years)**	76.7
**Age, range (years)**	21-100
**Mobility status (n):**	
**Immobile (n)**	87
**Reduced mobility (n)**	186
Mobile (n)*	106

*home visit for optimization of treatment

Table 2 shows the rates of the home visits for all considered quarter years, starting four quarter years before the beginning of the AGnES-project. The results of the statistical comparisons between the time periods before and during the AGnES-project are shown in Table 3.

**Table 2:** Basic data on the number of home visits from the ambulatory health care centre and the whole county Oberspreewald-Lausitz. The project started in the quarter year 3/2006

Time period	3/2005-2/2006			3/2006-2/2008		
	**Mean**	**St. dev**.	**Median**	**Mean**	**St. dev**.	**Median**

	**Ambulatory healthcare centre, all statutory insured patients**					

**Number of all GP-home visits/1000 patients**	168.2	15.7	173.1	132.9	23.8	136.0
**Number of usual GP-home visits/1000 patients**	131.3	13.4	134.6	106.8	26.3	113.0
**Number of medically urgent GP-home visits/1000 patients**	36.9	3.9	35.8	26.1	6.8	25.0
**Number of AGnES-home visits/1000 patients**	-	-	-	63.2	27.2	71.9
**Total number of home visits/1000 patients**	168.2	15.7	173.1	196.0	32.4	187.6

	**Ambulatory healthcare centre, AOK-patients**					

**Number of all GP-home visits/1000 AOK-patients**	216.6	26.2	223.0	159.0	47.6	152.9
**Number of usual GP-home visits/1000 AOK-patients**	171.5	23.9	171.7	132.1	45.4	122.4
**Number of medically urgent GP-home visits/1000 AOK-patients**	45.1	7.8	43.4	26.9	9.8	26.6
**Number of AGnES-home visits/1000 AOK-patients**	-	-	-	62.6	27.1	64.9
**Total number of home visits/1000 AOK-patients**	216.6	26.2	223.0	221.6	49.7	213.5

	**County Oberspreewald-Lausitz, AOK-patients**					

**Number of all GP-home visits/1000 AOK-patients**	138.8	11.0	134.9	134.5	6.3	133.8
**Number of usual GP-home visits/1000 AOK-patients**	128.4	10.8	124.2	124.6	5.9	124.8
**Number of urgent GP-home visits/1000 AOK-patients**	10.5	0.6	10.3	9.9	1.7	9.1

**Table 3:** Results of the comparison of the different types of home visits before and during the AGnES-project in the Federal State of Brandenburg

	Total number of home visits (GPs and AGnES-practice assistants)	Total number of GP-home visits	Number of usual GP-home visits	Number of medically urgent GP-home visits
**ambulatory healthcare centre, all statutory insured patients**				

**Z**	-1.529	2.208	1.698	2.208
**p**	0.126	0.027	0.089	0.027

**ambulatory healthcare center, AOK-patients**				

**Z**	0.170	1.868	1.698	2.378
**p**	0.865	0.062	0.089	0.017

**county Oberspreewald-Lausitz, AOK-patients**				

**Z**	-*	0.679	0.170	1.019
**p**	-*	0.497	0.865	0.308

Z: normally distributed z-value related to the calculated test statistic of the Wilcoxon rank-sum test

p: probability of error

* Since there were no AGnES-practice assistants in the surrounding county Oberspreewald-Lausitz, the comparison was only conducted for the GP-home visits.

### Analysis of the reimbursement data of all statutory insured patients of the ambulatory healthcare centre

**Figure 1:**
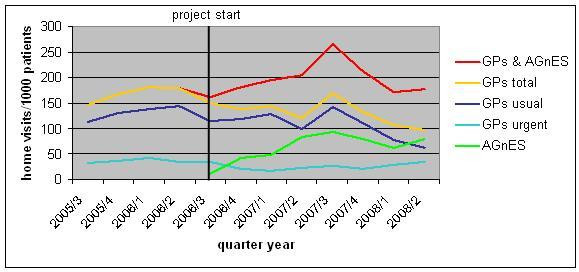
**Development of the number of GP- and AGnES-home visits of the ambulatory healthcare centre (all statutory insured patients)**.

Figure 1 shows the development of the home visit rates for all statutory insured patients of the ambulatory healthcare centre. The mean rate of the total number of home visits (sum of visits conducted by GPs and AGnES-practice assistants) with reference to all statutory insured patients was 168.2/1000 patients over the time period before the AGnES project started. During the project, the mean rate for the total number of home visits increased slightly to 196.0 home visits/1000 patients, this difference was not statistically significant (p = 0.126).

The home visit rate of the GPs decreased from 168.2 to 132.9 home visits/1000 patients. The difference for all statutory insured patients was statistically significant (p = 0.027). The most important effect arose from the decrease of urgent home visits (p = 0.027) (Table 3).

### Analysis of the data of the statutory health insurance AOK Brandenburg

#### Ambulatory healthcare centre

The average rate of GP-home visits over the four quarter years before the start of the project was 216.6 home visits per 1000 AOK-patients. In the eight quarter years during the project, the mean total rate of home visits (sum of visits by the GPs and AGnES-practice assistants) was 221.6. The trends for the home visits considering the AOK-patients are similar to the results for all statutory insured patients. In contrast to the home visits considering all statutory insured patients, this difference was not statistically significant (Wilcoxon rank-sum test; Z = 0.170, p = 0.865).

The number of home visits with AOK-patients by AGnES-practice assistants increased during the project from 11.3 (3. quarter year 2006, first quarter year of the project) to 87.6 home visits per 1000 AOK-patients (2nd quarter year 2008). The mean number of home visits during this period of time was 62.6 per 1000 AOK-patients.

Parallel to this increase in the delegated home visits (green curve in Figure 1) home visits conducted by the GPs decreased (yellow curve in Figure 1) from a mean value of 216.6 home visits per 1000 AOK-patients during the four quarter years before the start of the project to 159.0 home visits per 1000 AOK-patients during the time period of the project (Wilcoxon rank-sum test; Z = 1.868, p = 0.062).

The decrease of GP-home visits was more pronounced in the subgroup of medically urgent GP-home visits (light blue curve in Figure 1). This rate significantly decreased from 45.1 home visits/1000 AOK-patients before the project to 26.9 home visits/1000 AOK-patients during the project (Wilcoxon rank-sum test; Z = 2.378, p = 0.017).

#### County Oberspreewald-Lausitz

**Figure 2:**
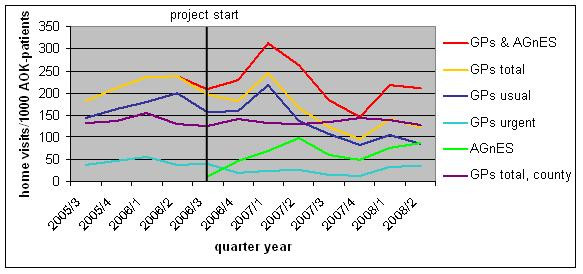
**Development of the number of GP- and AGnES-home visits of the ambulatory healthcare centre and the county Oberspreewald-Lausitz (AOK-patients)**.

None of these trends was visible in the surrounding county Oberspreewald-Lausitz. Here the home visit rates were more continuous (purple curve in Figure 2) and the temporal patterns were approximately equal for all types of GP-home visits (usual, urgent and total).

## Discussion

The analysis of the data of the ambulatory healthcare centre and the whole county in which the health care centre is located, describes the effects of introducing the delegation of GP-home visits to qualified praxis staff as a new option for the total system of home visits in this centre.

The study size was determined by the monocentric intervention and the county based organizational structure of the reimbursement data.

A limitation of the study is the absence of a prospective power calculation with respect to the development of the number of home visits. At the start of the project, we didn't know how many and which patients the GPs would select for this project, which activities would be delegated, the acceptance of the concept by the patients, and if the quality of care would be sufficient. In this context, a power calculation didn't seem to be useful.

The analysis is important to asses the eligibility of the AGnES-concept for implementing it into the regular health care system: a significant increase of the total number of home visits would have implied that the AGnES concept induced additional home visits in this setting. AGnES would then generate another financial burden on the statutory health insurances, which would increase the hesitation to add AGnES-home visits as a new option to their reimbursement catalogue.

An increase of the total number of home visits would only be acceptable if a region was actually underserved at the beginning of the project. In this case, the delegation of GP-home visits would provide a certain compensation for the lack of GP resources. As the analysed region was not underserved at the time of the project, an increase of home-visits would have indicated that the AGnES-option generates an additional demand of home visits either for a larger number of patients and/or to a higher frequency. The observation of a constant number of home visits before and during the intervention however, indicates that the new option had no major impact on the overall demand.

The results of this analysis show a clear trend towards a redistribution of home visits from the GP to qualified staff in his practice-team. There is an isolated peak in the curve of the GP-home visits of the ambulatory healthcare centre in the first quarter year of the year 2007 which is rather not associated with the project implementation, because it disappears already in the 2nd quarter year of the year 2007 while the frequency of the AGnES-home visits increases steadily until 2nd quarter year of the year 2007 (Figure 1).

Numerically the most extended effect is related to the medical urgent home visits. In the reimbursement catalogue, urgency is defined by date and time: home visits that have to be conducted after 7 pm, on bank holidays or during regular practice consultation-hours are defined as urgent and can be reimbursed with a higher remuneration. Hence practically, our results mean that the AGnES-concept enabled the GPs to reduce unscheduled home visits, thus avoiding working at unfavourable times and interruptions of regular consultation hours.

The setting of an ambulatory healthcare centre allowed performing an analysis of both the impact on the specific institution and on the regional population. Another advantage was the real time accessibility of the data used for reimbursement claiming, which were continuously documented with high quality. For the AGnES-practice assistants the complete information for each home visit was available from the standardized computer-assisted online documentation. The documentation of all AGnES-home visits on a patient-based level in a standardized computer-assisted documentation system assured high validity of this dataset.

However, this analysis has some limitations:

There is a difference between the subpopulation of the AOK-patients of the ambulatory healthcare centre and the AOK-patients in the county Oberspreewald-Lausitz: the basic population of the county consists of all AOK-patients which have their primary place of residence here. In Germany, patients are free to choose their GP and - depending on availability - in principle may change GPs whenever they want. Therefore, only patients who had at least one GP-contact in the respective quarter year were documented as patients of the ambulatory healthcare centre. The consequences can be lower home visit rates for the county, because here all patients, including those without a GP-contact in the respective period, are counted. The proportion of patients, who do not see a GP over a period of two consecutive years, however, is small [16].

Second, there is only a limited comparability between the total population of statutory insured patients of the ambulatory healthcare centre and the populations of AOK-patients of the healthcare centre and the surrounding county, because of large differences between the patients of the different German statutory health insurances, e.g. in age distribution, income, education, and profession.

Reimbursement data are collected primarily for reimbursement purposes and do not necessarily provide an exact picture of the actual medical activities. For example, urgent GP-home visits are defined by the time of the day. It is assumed that the GP will only make a home visits outside practice consultation-hours or at night if there is urgent medical need.

However, "home visit" constitutes a unit of reimbursement independent of its particular indication. All home visits are assigned to a specific institute. Hence, the assessment is likely complete and assumes a high validity of this dataset.

For these analyses, not the absolute numbers are important but rather the trends during the study period. It is unlikely and there is no indication that major changes happened over the study period.

Within the reimbursement data set, there was no specification for the gender of the patient and only a broad classification for age (≤5, 6-59, ≥ 60 years). Information about the medical condition of the patients was not available.

The changing conditions of primary care in Germany necessitate a change in the role of the GP from the solitary player of the past to the manager of a competent practice team. The future GP will distribute work packages flexibly to each member of his team, individually considering the specific competences necessary to perform them. Different concepts are being evaluated, e.g. the integration of practice nurses in the chronic care model [17] or in case management models for specific indications, e.g. heart failure [18]. The patients' acceptance of such new models of organizing primary care is generally quite good [13,14]. The implementation of innovative concepts into usual care, however, requires more than proven performance, good quality, and acceptance of the patients. Presently all participants in this process, physicians, nurses, and practice assistants, are still organized in separate traditional professional institutions, which for decades have focused on defining specific tasks and competences to maintain mutually exclusive professional spheres. Hence the main goal of the past was a perpetuation (and wherever possible extension) of professional borders and privileges, the future will focus on flexible work share. While any medical task as ever before requires the utmost responsibility and quality, the key issue will become qualification rather than profession. Since maintaining the quality of care is an inevitable condition for flexible work share concepts, the evaluation of objective parameters is an important issue. Regarding the AGnES-project, we are analysing different objective parameters (e.g. health related quality of life and the development of blood pressure values of patients with hypertension) to get objective indications for the quality of care of this concept.

Along this way, the traditional who-does-what-question in medical care will be fundamentally reconciled. We should put all effort on recruiting motivated staff, improve education, develop and provide flexible, evidence-based qualification and evaluate quality of care for patient-oriented results to meet the ever increasing demands of a growing, and aging number of patients in the next decades.

## Conclusions

Implementation of the AGnES-concept in an ambulatory healthcare centre in the Federal State of Brandenburg did not increase the total number of home visits. Rather, the delegation of home visits to AGnES-practice assistants reduced home visits previously conducted by the GP.

These results indicate that GPs can be effectively supported by AGnES-practice assistants without generating additional demand in the healthcare system.

In April 2009, the AGnES-concept was implemented into usual care for areas with an imminent or already existing undersupply with GPs. As the reimbursement and qualification requirements for the practice assistants were different from those found in the model projects, an evaluation under the conditions of usual care is necessary [19].

## Competing interests

The authors declare that they have no competing interests.

## Authors' contributions

NvdB drafted the manuscript and performed the statistical analysis. NvdB, CM, RH, MM, SF and WH participated in the design of the study. WH and CM helped to draft the manuscript. All authors read and approved the final manuscript.

## Pre-publication history

The pre-publication history for this paper can be accessed here:

http://www.biomedcentral.com/1472-6963/10/155/prepub
